# Modulation of cognitive and emotional processing by cannabidiol: the role of the anterior cingulate cortex

**DOI:** 10.3389/fnhum.2013.00147

**Published:** 2013-04-18

**Authors:** Mikael A. Kowal, Arno Hazekamp, Lorenza S. Colzato, Henk van Steenbergen, Bernhard Hommel

**Affiliations:** ^1^Cognitive Psychology Unit, Leiden Institute for Brain and Cognition, Leiden UniversityLeiden, Netherlands; ^2^Bedrocan B.V.Veendam, Netherlands

## Introduction

*Cannabis sativa* is a plant containing over 70 active compounds called cannabinoids (Schoedel and Harrison, 2012). The psychoactive effects of cannabinoids are abused worldwide by about 20% of young people, who report regular or heavy use of the cannabis plant (Moore et al., 2007). Delta-9-tetrahydrocannabinol (THC), the most prevalent cannabinoid in the plant, has been found to be responsible for producing most of the desirable effects of marijuana (Gaoni and Mechoulam, 1964). In line with that, the use of modern hydroponic cannabis farms has resulted in growing strains containing higher levels of THC, while keeping other cannabinoids at negligible levels (Hardwick and King, 2008). Accordingly, it may be assumed that the presence of THC-dominated cannabis plants on the market leads to the risk of more severe consequences of abuse, since THC has been associated with induction of psychotic symptoms both in an acute intoxicated state (D'Souza et al., 2004), as well as in the long-term (Kuepper et al., 2010). Consequently, in the current paper we propose that cannabidiol (CBD), another abundant compound of cannabis, might have an impact on cognition and emotional processing, which is opposite to the effect of THC. Moreover, we suggest that the effects of CBD would be worth investigating in regard to the modulatory role of the anterior cingulate cortex (ACC)—a brain region where both affective and cognitive information converge (Bush et al., 2000; Botvinick et al., 2001; Paus, 2001).

The pharmacology of CBD is well studied [for a review see Mechoulam et al. (2002)]. Its effects are distinct and frequently opposite to those of THC (Fusar-Poli et al., 2009). Whereas THC is a cannabinoid receptor type 1 and 2 (CB1r and CB2r) agonist, CBD has low affinity and a partially antagonistic effect at these receptors (Pertwee, 2008). Furthermore, CBD has been shown to be a serotonin receptor (5-HTr) agonist (Campos and Guimarães, 2008; Zanelati et al., 2010; Gomes et al., 2011). In recent years CBD has received renewed attention from researchers, mainly due to its anxiolytic (e.g., Zuardi et al., 1982, 1993; Crippa et al., 2004, 2011; Fusar-Poli et al., 2009; Bergamaschi et al., 2011) and antipsychotic effects (e.g., Zuardi et al., 2009; Bhattacharyya et al., 2010; Schubart et al., 2011). The therapeutic value of CBD in clinical contexts is currently being explored (Zuardi et al., 2006, 2009; Hallak et al., 2009). Moreover, in a recent review Schier et al. (2012) suggested that CBD neither produces psychoactive effects, nor has an impact on cognition. In the light of up-to-date research, this claim may be considered unwarranted, since CBD has been shown to differ with THC in terms of activation of brain regions during tasks involving response inhibition (Borgwardt et al., 2008), emotional processing (Fusar-Poli et al., 2009) and verbal memory (Bhattacharyya et al., 2010). Additionally, as far as only the anxiolytic effect of CBD is considered, it may be assumed that it influences cognition through, for instance, reducing attention bias toward threatening stimuli (Bar-Haim et al., 2007). In spite of that, the effect of CBD on cognitive performance has been largely unexplored.

## Effect of CBD on the ACC

CBD is associated with increased resting cerebral regional blood flow (rCBF) in the left parahippocampal gyrus and decreased rCBF in the amygdala-hippocampus complex, including the posterior cingulate cortex (Crippa et al., 2004). A functional neuroimaging (fMRI) study found evidence for attenuation of the blood-oxygen level dependent (BOLD) signal in the amygdala and the posterior and ACC in response to the presentation of fearful faces, combined with a reduction in subjective anxiety (Fusar-Poli et al., 2009). CBD also disrupts the functional connectivity between the ACC and amygdala (Fusar-Poli et al., 2010). Taken together, these results point to both an anxiolytic effect of CBD and a critical modulatory role of the ACC. However, Bhattacharyya et al. (2010) found no effect of CBD on ACC activity in a task identical to the one used by Fusar-Poli et al. (2009)—a discrepancy we will be getting back to. To summarize, apart from the emotion-regulating properties of CBD, the CBD–ACC relationship has not been systematically investigated.

Accordingly, it would be worthwhile to examine how the impact of CBD on ACC activity may extend to the domain of cognitive performance. Since modulation of ACC activity is assumed to be the mechanism through which CBD affects brain connectivity during emotional processing (Fusar-Poli et al., 2010), it might be suspected that the ACC is also the main target for CBD in terms of potential cognition-altering effects of the compound. Previous research has identified the ACC as an important relay station for cognitive control processes and as a region that integrates cognitive and emotional information (Bush et al., 2000; Botvinick et al., 2001; Paus, 2001). It is then possible that CBD has an effect on conflict monitoring - a process which monitors for the presence of conflicts in information processing. Conflict monitoring exerts top-down control over information processing by focusing attention on task-relevant processing streams, while blocking off task-irrelevant channels (Botvinick et al., 2001). In line with that, positron emission tomography (PET) and fMRI studies reliably show ACC activation in tasks in which subjects need to override automatic, but otherwise task-irrelevant responses, such as the Stroop Color Word Test (SCWT; Pardo et al., 1990; Carter et al., 1995; Bush et al., 1998) and go/no-go tasks (Kawashima et al., 1996; Casey et al., 1997). Since CBD has been shown to decrease activity in the ACC (Fusar-Poli et al., 2009, 2010), it may be suspected that individuals treated with CBD are less likely to suppress their dominant response (Casey et al., 1997), or become aware of committing a mistake (Holroyd and Coles, 2002).

## Effect of CBD on cognition

Taken together, research regarding the impact of CBD on ACC activity appears to be contradictory (see Table 1): CBD has been reported to attenuate ACC activity (Fusar-Poli et al., 2009, 2010), have no effect (Borgwardt et al., 2008; Bhattacharyya et al., 2009, 2010), or even enhance ACC activity (Bhattacharyya et al., 2010). One possible explanation for these contradictory observations may be found in the type of tasks used in the studies mentioned and, thus, the functions which they relate to. In the case of cognition, keeping in mind the involvement of the ACC in conflict monitoring (Bush et al., 2000; Botvinick et al., 2001; Paus, 2001), it would be worthwhile to have a closer look at the findings of Bhattacharyya et al. (2010). The observed increased activity of the ACC during a verbal recall task is in line with research showing lack of impairment of verbal memory in cannabis users intoxicated with high-CBD content cannabis (4.61% on average), as opposed to those that used low-CBD content plant material (0.08% on average; Morgan et al., 2010). Furthermore, it has been suggested that the memory-protective effect of CBD extends into the long-term (Morgan et al., 2012). Combining the results of the above-mentioned studies, one could claim that the CBD-induced improvement is not restricted to the domain of memory itself, but reflects a more general enhancement of the conflict monitoring system. Additionally, such a claim becomes somewhat more plausible when one takes into account a recent investigation which found a trend for decreased response latency to oddball stimuli following CBD administration together with, surprisingly, an attenuating effect on the medial prefrontal cortex (Bhattacharyya et al., 2012). Although this opposite effect of CBD on activation and the finding of no effect of CBD on ACC activity in another study applying the verbal recall task make the case less clear (Bhattacharyya et al., 2009), it is possible that the beneficial effect of CBD is more visible in case of modulating the deteriorating effects of THC than when administered alone. Since THC is a CB1r and CB2r agonist, the opposite, partially antagonistic, effect of CBD on CB1rs and CB2rs suggests that it may protect against the deterioration of cognitive performance caused by THC (Pertwee, 2008). THC has been shown to decrease the error-related negativity—an event-related potential indicative of conflict monitoring and assumed to be generated by the ACC (Spronk et al., 2011). Given the opposing neuropharmacological actions of the two compounds, one may then expect that CBD will inhibit the impact of THC on the ACC. On the other hand, in the absence of THC, partial antagonism of CB1rs might not be sufficient to produce overt changes in the conflict monitoring system, either at the behavioral or the neurophysiological level. Accordingly, it would be worthwhile to explore whether the THC-protective effects of CBD can be related directly to ACC functioning.

**Table 1:** **Functional MRI studies of the cognitive and emotional effects of cannabidiol**.

	**Sample size**	CBD dose^***^	**Route**	**Task**	**ACC activation**	**Amygdala activation**	**Anxiolytic effect**
**COGNITION**							
Borgwardt et al. (2008)	15	600 mg	Oral	Response inhibition (go/no-go)	*n.s*.	*n.s*.	*p* = 0.06
Bhattacharyya et al. (2009)	15	600 mg	Oral	Verbal paired associate learning	*n.s*.	*n.s*.	*n.s*.
Bhattacharyya et al. (2010)^*^	15	600 mg	Oral	Verbal paired associate learning	*p* < 0.01	*n.s*.	*p* = 0.06
**EMOTION**							
Fusar-Poli et al. (2009)	15	600 mg	Oral	Facial expressions of emotion: stimuli and tests	*p* < 0.05	*p* < 0.05	*p* = 0.06
Bhattacharyya et al. (2010)^*^	15	600 mg	Oral	Facial expressions of emotion: stimuli and tests	*n.s*.	*p* < 0.05	*p* = 0.06
Fusar-Poli et al. (2010)^**^	15	600 mg	Oral	Facial expressions of emotion: stimuli and tests	*p* < 0.05	*p* < 0.05	n/a

* *Contrasted with effects of delta-9-tetrahydrocannabinol (10 mg, 99.6% pure)*.

** *Dynamic causal modeling—significant activation indicating a difference in the forward intrinsic connection between ACC and amygdala*.

*** *99.9% pure in all studies*.

## Effect of CBD on affective processing

It is also interesting to consider the ACC-mediated impact of CBD on emotional processing. Animal research indicates that the anxiolytic effect of CBD depends on action of the compound on specific brain areas and that the effect could also, in some cases, be anxiogenic (Marco et al., 2011). In case of human studies, it can be hypothesized that CBD decreases ACC activity, which would be in line with the anxiolytic effect of the compound (Fusar-Poli et al., 2009). The concurrent reduction in amygdala BOLD response associated with presentation of fearful faces gives further support to this assumption, given the involvement of this region in fear processing and its anatomical connection with the ACC (Fusar-Poli et al., 2009, 2010; Bhattacharyya et al., 2010). However, while both Fusar-Poli et al. (2009) and Bhattacharyya et al. (2010) found a trend in reduction of subjective anxiety following CBD administration, only the former was able to observe a related decrease in ACC activation during emotional processing. In case of the latter, the authors explain the lack of a possible effect by a selective analysis of brain areas where THC and CBD had opposite effects, instead of assessing the effects of the two compounds separately (Bhattacharyya et al., 2010). However, the fact that application of the same design, task and subject sample led to different results throws some doubt on the importance of the ACC in mediating the effects of CBD on brain connectivity (Fusar-Poli et al., 2010). Moreover, if one were to follow the logic of Fusar-Poli et al. (2010) about top-down control of the ACC over the amygdala, attenuation of the amygdala BOLD response in the Bhattacharyya et al. (2010) study should not have been observed without a simultaneous effect in the ACC. In principle, the anxiolytic effects of CBD are assumed to be mediated by the ACC, which, in turn, affects activity of the amygdala (Fusar-Poli et al., 2010). Therefore, from this perspective, it is surprising that the reduction in subjective anxiety observed by Bhattacharyya et al. (2010) was indeed associated with a concurrent decrease in amygdala activation, but not the ACC. If the ACC actually plays a critical role in moderating the anxiolytic effects of CBD (Fusar-Poli et al., 2010), then it seems plausible to expect an effect in this brain region, even when keeping in mind the selective analysis of opposing effects of THC and CBD on activation (Bhattacharyya et al., 2010). On the other hand, the absence of an effect of CBD may alternatively be explained by the lack of a significant linear relationship between the effects of the two drugs and placebo. Consequently, the fact that Bhattacharyya et al. (2010) did not find CBD to be associated with decreased ACC activity, is not necessarily equivalent to the lack of an effect of CBD relative to a placebo condition. In any case, apart from evidence pointing to a link between CBD and the ACC in emotional processing, it is likely that this connection is not as straightforward as suggested by Fusar-Poli et al. (2010).

## Summary

In sum, existing research seems to undermine Schier's et al. (2012) suggestion regarding the lack of a relationship between CBD and cognition. Rather, it seems that both cognitive and affective consequences of CBD administration may be mediated by the ACC. However, the lack of clear-cut results renders the extent and nature of this modulation unclear. The diversity of findings may be explained by various factors, including differential activation of the cognitive and affective subdivisions of the ACC (Bush et al., 2000), the slow onset of action and inconsistent bioavailability of orally administered cannabinoids (Hazekamp et al., 2006), the dosage of CBD (Marco et al., 2011), or whether CBD was administered alone, or in combination with THC (Pertwee, 2008). Furthermore, since modulation of the cognitive effects of cannabinoids has been linked to polymorphism of the cannabinoid receptor (CNR1) gene (Beng-Choon et al., 2011; Stadelman et al., 2011), it is possible that some genetic predispositions of the studied samples could have influenced the results. From this perspective, also the catechol-O-methyltransferase (COMT) gene seems to be a plausible moderating factor due to its role in cognitive control (Colzato et al., 2010) and the pharmacological interactions between the endocannabinoid and dopamine systems (Fattore et al., 2010). In any case, inclusion of new variables could further clarify the CBD–ACC relationship and its role in the aspects of conflict monitoring and emotional processing.
